# Efficacy of radiotherapy in primary mediastinal diffuse large B-cell lymphoma: a systematic review and meta-analysis of 1,392 patients

**DOI:** 10.1007/s00277-025-06530-8

**Published:** 2025-09-25

**Authors:** Dongyu Zhuang, Silan Huang, Peng Zhang, Dexin Lei, Yanlou Wang, Honglian Liu, Man Nie, Yi Xia

**Affiliations:** Department of Medical Oncology, State Key Laboratory of Oncology in South China, Guangdong Provincial Clinical Research Center for Cancer, Collaborative Innovation Center for Cancer Medicine, Sun Yat-sen University Cancer Center, Guangzhou, Guangdong People’s Republic of China

**Keywords:** Lymphoma, Primary mediastinal b-cell lymphoma, Radiotherapy, Meta-analysis

## Abstract

Primary mediastinal B-cell lymphoma (PMBCL) patients who achieve a complete response (CR) following first-line immunochemotherapy tend to have favorable outcomes, even without subsequent radiotherapy (RT). However, it remains unclear whether the survival of patients who do not achieve CR after first-line therapy and subsequently receive radiotherapy is comparable to that of CR patients who undergo observation alone. To address this gap, we conducted a systematic review and meta-analysis to compare progression-free survival (PFS) and overall survival (OS) between CR patients who forgo radiotherapy and non-CR patients who receive consolidative radiotherapy. Additionally, we examined potential influencing factors to provide evidence-based guidance for clinical treatment strategies. A comprehensive search of multiple databases (PubMed, EMBASE, and Cochrane Library) was conducted for the period from January 1, 2012, to September 1, 2024. Using predefined keywords and screening procedures, 15 studies were ultimately included. The primary endpoint was PFS, with OS as a secondary endpoint. Subgroup analyses were performed based on chemotherapy regimens. This systematic review and meta-analysis of 15 studies demonstrated that consolidative radiotherapy significantly improves PFS in patients with partial remission (PR) after first-line immunochemotherapy, with an overall risk ratio (RR) of 1.11 (95% confidence interval [CI]: 1.05–1.16). For OS, the RR was 1.04 (95% CI: 0.97–1.12), crossing the line of no effect, which suggests that consolidative radiotherapy does not have a statistically significant impact on OS. Consolidative radiotherapy improves PFS in patients with PMBCL, but its effect on OS is not statistically significant.

## Introduction

Primary mediastinal large B-cell lymphoma (PMBCL) is a relatively rare lymphoma, initially defined as a subtype of diffuse large B-cell lymphoma (DLBCL) in the revised European-American classification of lymphoid neoplasms, based on its distinct clinical and pathological characteristics [1]. However, the majority of PMBCL cases show CD30 positivity at the pathological level, and recent molecular profiling studies have revealed significant genetic expression similarities between PMBCL and classical Hodgkin’s lymphoma (cHL) [2]. More than one-third of genes highly expressed in PMBCL are also characteristically expressed in cHL cells. Based on these findings, the World Health Organization (WHO) now recognizes PMBCL as a distinct tumor entity [3].

Rituximab-based chemoimmunotherapy is widely regarded as the standard first-line treatment for PMBCL [4–6]. Standard regimens include rituximab, cyclophosphamide, doxorubicin, vincristine, and prednisone (R-CHOP) or its variants, typically followed by consolidative mediastinal RT [7, 8]. Early studies reported 5-year PFS rates of 77–81% and OS rates of 84–89% in patients treated with R-CHOP plus RT [4, 9, 10]. However, the impact of mediastinal RT on PFS and OS remains controversial, as retrospective studies have produced inconsistent results [11–13]. Furthermore, some analyses have suggested that even without consolidative RT, the dose-adjusted R-EPOCH (Da-R-EPOCH) regimen achieves PFS and OS outcomes comparable to those of R-CHOP plus RT [14–16]. The advent of PET-CT imaging has facilitated response-adaptive treatment strategies, aimed at minimizing unnecessary RT exposure.

To investigate the role of RT in patients achieving CR, the phase III IELSG37 trial employed PET-CT to assess CR status following first-line chemotherapy. Patients who achieved CR were randomized to receive either RT or observation. After a median follow-up of 30 months, the 5-year PFS rates were 98.5% and 96.2% for the RT and observation groups, respectively, with no statistically significant difference between the two groups. These findings suggest that for patients achieving CR after first-line chemoimmunotherapy, omitting RT does not compromise prognosis. In contrast, for patients who did not achieve CR, the 5-year PFS and OS rates were 60.3% and 74.6%, respectively. However, only 57% of these patients received RT, leaving the question of whether RT can improve survival in patients who did not achieve CR to levels comparable to those of CR patients unresolved. Currently, there is a lack of systematic statistical analyses on the prognosis of patients who did not achieve CR, and further comprehensive studies are needed to determine whether consolidative RT can yield therapeutic outcomes comparable to those of CR patients.

Therefore, this study aims to systematically analyze published data on first-line treatment for PMBCL, focusing on patients who achieved CR with regular follow-up, as well as those who did not achieve CR but received consolidative RT. By comparing PFS and related outcomes between these groups, we aim to evaluate the necessity of consolidative RT in the treatment of PMBCL.

### Methods

1. Search strategy

We systematically searched PubMed, EMBASE, and Web of Science for studies published between January 1, 2012, and September 1, 2024. The starting point of 2012 was selected to align with the widespread adoption of PET-CT for treatment response assessment, ensuring consistency in outcome evaluation. The search terms included “lymphoma” or “radiotherapy,” combined with free-text terms such as “malignant,” “B-cell,” and “radiation therapy.”

#### Medical subject headings (MeSH) included

Lymphoma; free-text terms included: Lymphomas, Lymphoma, Malignant, Lymphomas, Malignant, Malignant Lymphoma, Malignant Lymphomas, Lymphoma, B Cell, Lymphoma, B-Cell, etc.

#### MeSH terms included

Radiotherapy; free-text terms included: Radiotherapies, Radiation Therapy, Radiation Therapies, Therapies, Radiation, Therapy, Radiation, Radiation Treatment, Radiation Treatments, Treatment, Radiation, etc.

To ensure the comprehensiveness of the search and adequate coverage of potentially relevant studies, we also reviewed the reference lists of identified articles and results from previous meta-analyses.

2) Inclusion and exclusion criteria

#### Inclusion criteria

A study was included if it met the following criteria: (1) Diagnosis of PMBCL was confirmed by pathology; (2) Treatment efficacy was evaluated using either CT or PET-CT after the completion of chemotherapy; (3) Participants were grouped based on the efficacy evaluation, including a radiotherapy consolidation group and an observation group; (4) The study reported essential information, such as the total number of patients, age, and chemotherapy regimen; (5) The study provided data on PFS and OS.

#### Exclusion criteria

Studies were excluded if they met any of the following conditions: (1) Incomplete data or inconsistent study design, such as unspecified sample size or lack of post-chemotherapy efficacy evaluation; (2) Data pertaining to pediatric PMBCL patients or PMBCL subgroup data not reported in published series studies; (3) Significant flaws in statistical methods or experimental design; (4) Case reports, studies with a sample size of fewer than 5 cases, and unpublished conference abstracts.

3) Data extraction

Based on the inclusion and exclusion criteria, two researchers (ZDY, ZP) independently and repeatedly extracted data using a pre-designed data collection form. Discrepancies were resolved by a senior researcher (NM) through coordination and discussion among the researchers.

The quality of prospective studies was independently assessed using the Cochrane Collaboration’s risk of bias tool (GRADE profiler software), while retrospective studies were evaluated using the Newcastle-Ottawa Scale (NOS). Only studies with a NOS score of ≥ 6 were deemed eligible for inclusion.

The extracted information included the first author, publication date, study type, total number of patients, chemotherapy regimen, PFS and OS data for both the observation group and radiotherapy consolidation group, follow-up duration, and other relevant details.

**Fig. 1:**
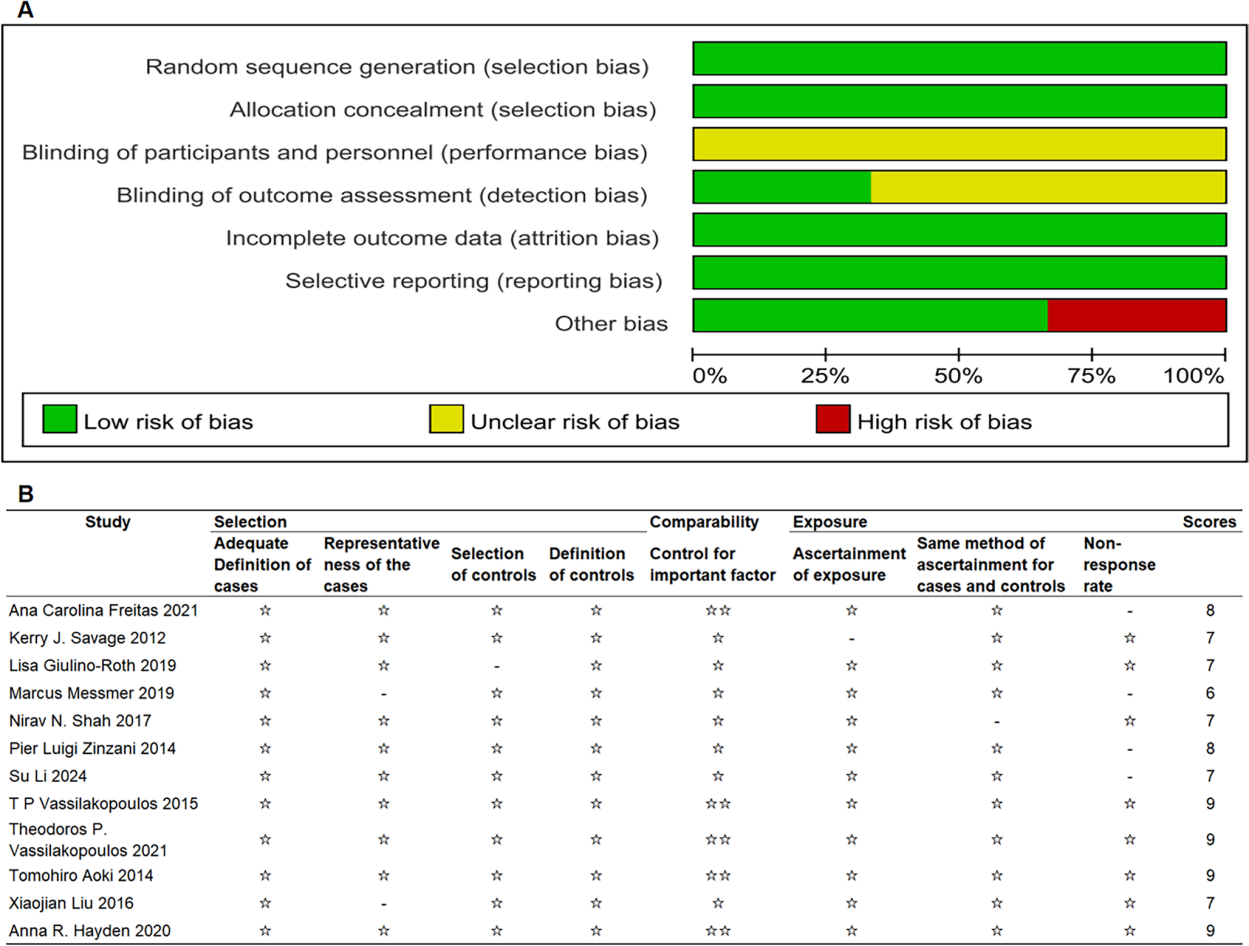
Quality assessment of included studies.** A**: Prospective studies were independently evaluated using the Cochrane Collaboration Risk of Bias Tool. **B**: Retrospective studies were assessed using the NOS. Scores of 7–9 were considered high-quality studies, scores of 4–6 were considered moderate-quality studies, and scores below 4 were considered low-quality studies

The data extraction process adhered to the **Preferred Reporting Items for Systematic Reviews and Meta-Analyses (PRISMA)** guidelines [17]. A summary of the quality assessment of included studies is presented in Fig. 1.

4) Outcome measures

The primary endpoint of this study was PFS, defined as the time from randomization (or treatment initiation in single-arm studies) to the first occurrence of tumor progression, last follow-up, or death. The secondary endpoint was OS, defined as the time from randomization (or treatment initiation in single-arm studies) to death from any cause.

5) Data analysis

The Kaplan-Meier (K-M) curves from the included studies were extracted using the **Digital** package in R software. The aggregated K-M curves were plotted and analyzed using the **MetaSurv** package. The version of R software used was 3.6.3. Heterogeneity was assessed using the H statistic, with H < 1.2 indicated non-significant heterogeneity.

Statistical analyses were performed using Review Manager (version 5.4/2021). The efficacy of observation versus radiotherapy after chemotherapy was evaluated by calculating PFS and OS, expressed as relative risks (RRs) with corresponding 95% confidence intervals. Although hazard ratios (HRs) are the preferred measure for time-to-event outcomes, most included studies did not report HRs or provide sufficient data for reconstruction. Accordingly, RR was employed to assess PFS and OS.

Heterogeneity among studies was further assessed using the chi-square test (Q statistic) and the I² statistic. If *P* ≥ 0.10 and/or I² < 50%, heterogeneity was considered low, and a fixed-effects model (Mantel–Haenszel method) was applied; otherwise, a random-effects model was used. Publication bias was assessed using the Begg and Egger tests, with a P-value < 0.05 indicating significant publication bias.

## Results

### Study characteristics and quality assessment

**Fig. 2:**
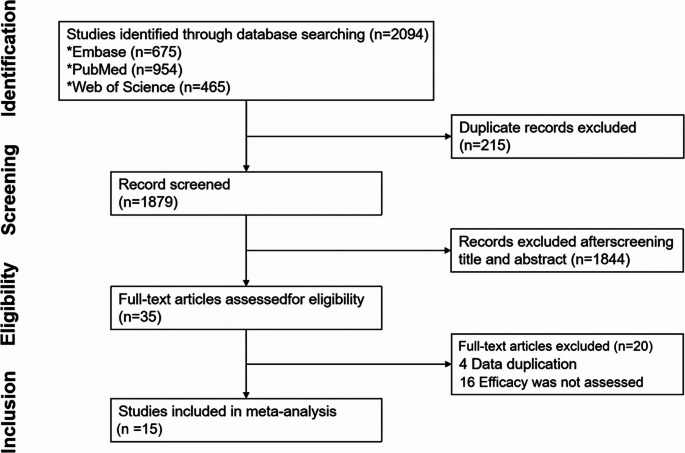
PRISMA flow chart of the study selection process. PRISMA, preferred reporting items for systematic reviews and meta-analyses

A total of 2,094 relevant studies were identified through the Literature search, of which 15 met the predefined inclusion criteria and were ultimately included in the analysis [18–32]. The detailed selection process is illustrated in Fig. 2. Among these, 3 were prospective studies and 12 were retrospective.

This meta-analysis included data from 1,392 adult patients with PMBCL who received first-line chemoimmunotherapy. Of these, 762 patients who achieved CR were assigned to the observation group, while 630 patients, who did not attain CR, underwent consolidative radiotherapy.

The median follow-up duration across studies was 49.4 months. Due to the unavailability of detailed demographic data for each subgroup in most studies, baseline characteristics were not summarized. Key information for the two treatment groups is presented in Table 1.

**Table 1:** Baseline characteristics and main outcomes of included studies with radiotherapy in PMBCL

	Region	Study type	Median follow-up months	Sample size	Drug	mPFS (95% CI) observation	mPFS (95% CI) consolidative radiotherapy	OS (95% CI) observation	OS (95% CI) consolidative radiotherapy
Ana Carolina Freitas 2021 [18]	Portugal	Retrospective study	67	37	R-CHOP	5-year PFS:100%	5-year PFS:66.67%	NR	NR
Anna R. Hayden 2020 [19]	Canada	Retrospective study	94.8	103	R-CHOP	5-year PFS: 90% [95% CI, 74–95%]	5-year PFS: 81% [95% CI, 72–87%]	5-year OS: 86% [95% CI, 73–93%]	5-year OS: 91% [95% CI, 83–95%]
Franco Cavalli 2024[20]	Europe	Open-label, multicenter, single-arm, phase 3	60	364	R-CHOP R-DA-EPOCH R-MACOP-B	5-year PFS: 86.6% [95% CI, 80.3–91.6%]	5-year PFS: 96.3% [95% CI, 93.4–97.9]	5-year OS: 92.85% [95% CI, 85.55–95.45%]	5-year OS: 99% [95% CI, 96.9–99.7%]
Gerhard Held 2023 [21]	German	Open-label, multicenter, single-arm, phase 3	120.2	51	R-CHOP	3-year PFS: 90% [95% CI, 81–98%]	3-year PFS: 95% [95% CI, 90–100]	3-year OS: 96% [95% CI, 90–100%]	3-year OS: 98% [95% CI, 94–100%]
Kerry J. Savage 2012 [22]	Canada	Retrospective study	64.8	56	R-CHOP	5-year PFS: 78%	5-year PFS: 83%	5-year OS: 88.5%	5-year OS: 95%
Lisa Giulino-Roth 2019 [23]	USA	Retrospective study	24	137	R-DA-EPOCH	2-year PFS: 68.8%	2-year PFS: 66.7%	NR	NR
Marcus Messmer 2019 [24]	USA	Retrospective study	36	26	R-CHOP	3-year PFS: 93.75%	3-year PFS: 100%	NR	NR
Maurizio Martelli 2014 [25]	Italy/UK	Open-label, multicenter, single-arm, phase 3	35	115	R-MACOP-B R-CHOP	5-year PFS: 97.06%	5-year PFS: 84.62%	NR	NR
Nirav N. Shah 2017 [26]	UK	Retrospective study	65	26	R-CHOP	2-year PFS: 88%	2-year PFS: 95%	NR	NR
Pier Luigi Zinzani 2014 [27]	Italy	Retrospective study	62	74	R-MACOP-B	10-year PFS: 90%	10-year PFS: 90.1%	NR	NR
Su Li 2024 [28]	UK	Retrospective study	66	32	R-CHOP	5-year PFS: 89.3%	5-year PFS: 75%	5-year OS: 96.3%	5-year OS: 75%
T P Vassilakopoulos 2015 [29]	Greece	Retrospective study	60	70	R-CHOP	5-year PFS: 92.86%	5-year PFS: 87.32%	NR	NR
Theodoros P. Vassilakopoulos 2021 [30]	Greece	Retrospective study	36	127	R-CHOP	3-year PFS: 93.75%	3-year PFS: 73.69%	3-year OS: 96.88%	3-year OS: 86.84%
Tomohiro Aoki 2014 [31]	Japan	Retrospective study	48	122	R-CHOP	4-year PFS: 77%	4-year PFS: 85%	4-year OS: 95%	4-year OS: 100%
Xiaojian Liu 2016 [32]	China	Retrospective study	60	52	R-CHOP	5-year PFS: 66.7%	5-year PFS: 70%	5-year OS: 58.3%	5-year OS: 67.5%

In most studies, patients were assessed for efficacy. Some studies were summarized in tables without 95% confidence intervals. In the other subset of studies, overall survival was not assessed. In studies reporting broader endpoints such as event-free survival (EFS) or freedom from progression (FFP), only events consistent with the definition of progression-free survival (PFS)—including disease progression, relapse, or death from any cause—were extracted. This composite measure is referred to as modified progression-free survival (mPFS) throughout the analysis. mPFS, modified progression-free survival; *CI*, confidence interval; *OS*, overall survival; *NR*, not reported; *R-DA-EPOCH*: rituximab, dose-adjusted etoposide, prednisone, vincristine, cyclophosphamide, doxorubicin; *R-MACOP-B*: rituximab, etoposide, doxorubicin, cyclophosphamide, vincristine, prednisone, bleomycin; *R-CHOP*: rituximab, cyclophosphamide, doxorubicin, vincristine, prednisone.

Regarding response assessment, only one study evaluated treatment response using CT following immunochemotherapy, in which responses were assessed according to the 1999 International Workshop Criteria [33]. Among the remaining 14 studies utilizing PET-CT for response evaluation, one [25] applied the International Harmonization Project (IHP) criteria [34], while the others adopted the Deauville five-point scale [35]. In these studies, PET-negative findings–interpreted as CR-corresponded to Deauville scores of 1 to 3 (D1-D3) or uptake attributed to alternative causes (DX), Whereas PET-positive results (D4-D5) were considered indicative of partial remission (PR), stable disease (SD), or progressive disease (PD). In some studies, only Deauville scores of 4–5 were reported without specifying whether the outcomes represented partial remission (PR) or progressive disease (PD). As a result, the objective response rate (ORR) data were missing in these studies.

Details regarding the radiation dose and field, as well as the interval between the final cycle of immunochemotherapy and the initiation of radiotherapy in each study, are summarized in Table 2.

**Table 2:** ORR, PET-CT assessment, and radiotherapy characteristics of included studies in PMBCL

Study	Dose-dense/ Dose-intensity	CRR%	ORR%	Grade ≥ 3 side effects%	Judging criteria for CR and PR	Interval between immunochemotherapy and irradiation	Radiotherapy dose
Ana Carolina Freitas 2021 [18]	R-CHOP-14:2% R-CHOP-21:98%	60%	88%	50%	PET-CT	NR	NR
Anna R. Hayden 2020 [19]	R-CHOP-21	71%	NR	NR	PET-CT	NR	NR
Franco Cavalli 2024[20]	R-CHOP-14:15.4% R-CHOP-21:26.8% R-DA-EPOCH-21:31.1% R-MACOP-B-21:16.0%	55%	NR	NR	PET-CT	within 8 weeks	Median 3000 cGy
Gerhard Held 2023 [21]	R-CHOP-14:46.6% R-CHOP-21:53.4%	90.1%	95.4%	56%	CT + PET-CT	2–6 weeks	Median 3960 cGy
Kerry J. Savage 2012 [22]	R-CHOP-21	59%	NR	NR	PET-CT	NR	NR
Lisa Giulino-Roth 2019 [23]	R-DA-EPOCH-21	75%	NR	NR	PET-CT	NR	Median 3640 cGy
Marcus Messmer 2019 [24]	R-CHOP-21	74%	98%	NR	CT + PET-CT	NR	NR
Maurizio Martelli 2014 [25]	R-CHOP-21:6% R-CHOP-14:6% Intensified R-CHOP:5% R-MACOP-B-21: 81%	47%	NR	NR	PET-CT	within 8 weeks	3000 to 4200 Gy
Nirav N. Shah 2017 [26]	R-CHOP-21	69.6%	75%	16.1%	PET-CT	NR	NR
Pier Luigi Zinzani 2014 [27]	R-MACOP-B-21	82.4%	NR	6.8%	CT + PET-CT	4–6 weeks	3000 to 3600 cGy
Su Li 2024 [28]	R-CHOP-14:28% R-CHOP-21:72%	72%	NR	NR	PET-CT	NR	NR
T P Vassilakopoulos 2015 [29]	R-CHOP-14:12% R-CHOP-21:88%	59%	NR	NR	PET-CT	median of 35 days	Median 4000 cGy vs. 3560 cGy (positive vs. negative)
Theodoros P. Vassilakopoulos 2021 [30]	R-CHOP-14:9% R-CHOP-21:91%	58%	NR	NR	PET-CT	NR	Median 3420 cGy vs. 3600 cGy vs. 4000 cGy vs. 4400 cGy (D5PSS-1/2 vs. 3 vs4 vs5)
Tomohiro Aoki 2014 [31]	R-CHOP-21	64.2%	91.4%	NR	CT	NR	NR
Xiaojian Liu 2016 [32]	R-CHOP-21	76.9%	78.9%	NR	CT + PET-CT	NR	Median 3600 cGy

*CRR*, complete response rate; *ORR*, objective response rate; *NR*, not reported; *CT*: computed tomography; *PET-CT*: positron emission tomography-computed tomography; *cGy*, centigray; *D5PSS*: Deauville 5-point scale; *R-DA-EPOCH*: rituximab, dose-adjusted etoposide, prednisone, vincristine, cyclophosphamide, doxorubicin; *R-MACOP-B*: rituximab, etoposide, doxorubicin, cyclophosphamide, vincristine, prednisone, bleomycin; *R-CHOP*: rituximab, cyclophosphamide, doxorubicin, vincristine, prednisone.

### Efficacy

**Fig. 3:**
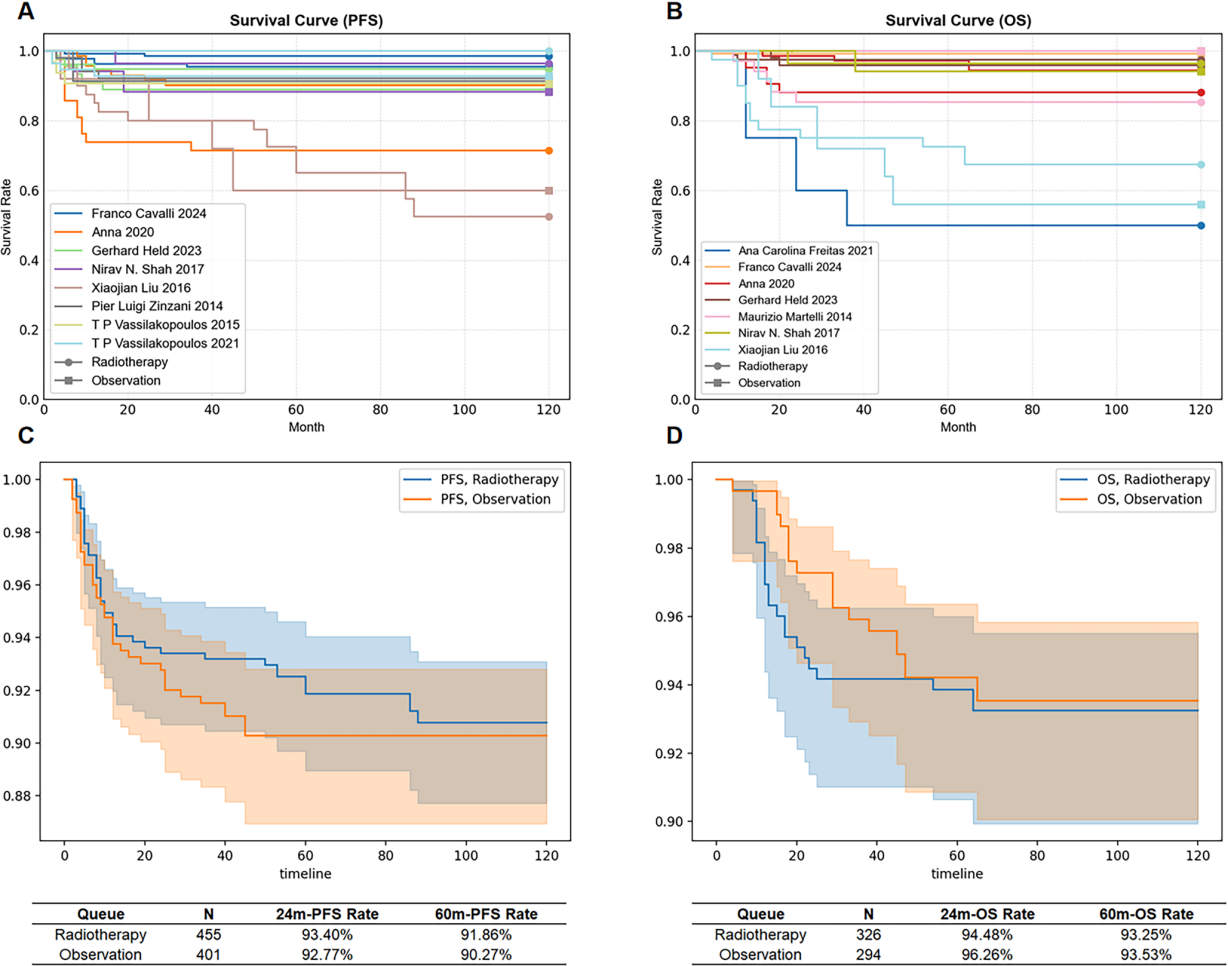
Pooled Kaplan–Meier estimate of PFS and OS. (**A**) PFS curves for total group; (**B**) OS curves for total group; (**C**) Combined PFS curve of the radiotherapy group and observation group; (**D**) Combined Kaplan–Meier curve for OS of the radiotherapy group versus observation group. *PFS,* progression-free survival; *OS,* overall survival; *CI,* confidence interval; 24 m-PFS: 24-month progression-free survival; 60 m-PFS: 60-month progression-free survival; 24 m-OS: 24-month overall survival; 60 m-OS: 60-month overall survival

Figure 3A and B illustrate the reconstructed Kaplan–Meier (K-M) curves for PFS and OS, respectively, generated by digitizing and aggregating data extracted from the published K-M curves of 8 and 7 eligible studies. For patients who did not achieve CR (non-CR) but received consolidative RT, the pooled estimated PFS rate was 93.40% at 24 months and 91.86% at 60 months (Fig. 3C). The pooled estimated OS rate was 94.48% at 6 months and 93.25% at 12 months (Fig. 3D). For patients who achieved complete remission after chemotherapy, the pooled estimated PFS rate was 92.77% at 24 months and 90.27% at 60 months (Fig. 3C). The pooled estimated OS rate was 96.26% at 24 months and 93.53% at 60 months (Fig. 3D). The P-values for the PFS and OS curves between the two cohorts were 0.78 and 0.84, respectively, neither of which demonstrated a statistically significant difference.

### Impact of consolidation radiotherapy on primary outcome (PFS)

**Fig. 4:**
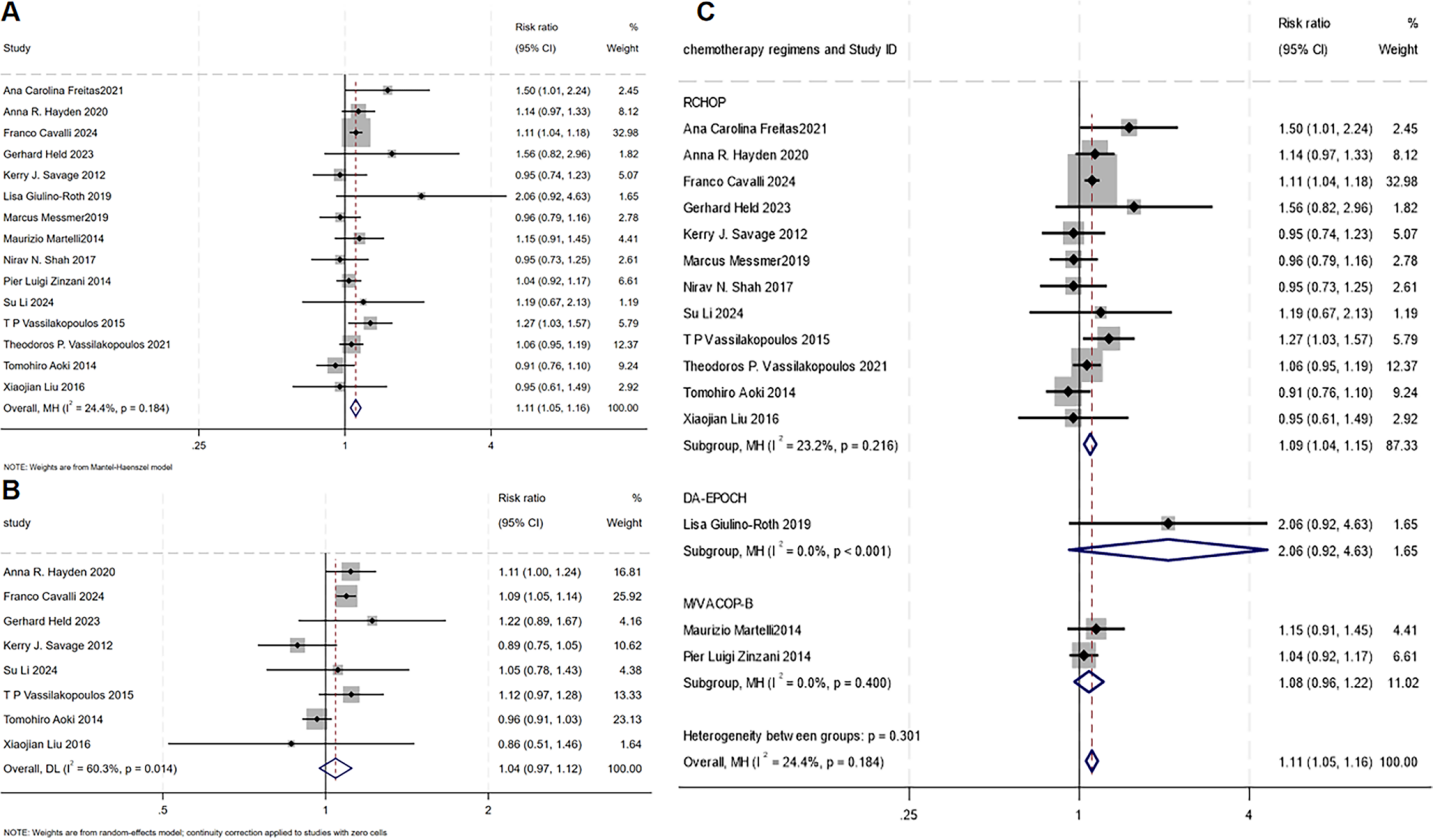
Summary results of overall PFS and OS, with subgroup comparisons of PFS based on drug regimens. (**A**) Forest plot of PFS for the two cohorts; (**B**) Forest plot of OS for the two cohorts; (**C**) Subgroup analysis stratified by chemotherapy regimen. Number of patients and events by treatment regimen: R-CHOP group (*n* = 863, events = 156); DA-EPOCH-R group (*n* = 137, events = 47); MACOP-B group (*n* = 145, events = 10). Note: The heterogeneity among studies was assessed using the Cochran Q chi-square test and I² statistic. A Q test with *P* < 0.1 was considered indicative of high heterogeneity, and an I² value > 50% was regarded as a marker of moderate-to-high heterogeneity. If the conclusions of the Q test and the I² statistic were contradictory, priority was given to the conclusion derived from the I² statistic, as the Q test has been shown to have limited power in detecting heterogeneity in previous studies

All 15 included studies evaluated the impact of consolidation radiotherapy on the PFS of the two cohorts. The pooled analysis results are shown in Fig. 4A. The overall risk ratio (RR) was 1.11 (95% confidence interval [CI]: 1.05–1.16), indicating a positive effect of consolidation radiotherapy on PFS.

Heterogeneity analysis showed low heterogeneity (I² = 24.4%, *P* = 0.184), and therefore, a fixed-effects Mantel-Haenszel method was applied. These findings suggest that consolidation radiotherapy significantly improves PFS in patients who did not achieve CR but received consolidative RT.

### Impact of consolidation radiotherapy on secondary outcome (OS)

Eight included studies assessed the impact of consolidation radiotherapy on OS of the two cohorts. The pooled analysis results are shown in Fig. 4B. Heterogeneity analysis revealed moderate heterogeneity (I² = 60.3%, *P* = 0.014), and thus a random-effects model was used for analysis.

The overall RR was 1.04 (95% confidence interval [CI]: 0.97–1.12). The analysis of secondary outcomes indicated that consolidation radiotherapy had no statistically significant effect on OS of patients in the two cohorts.

### Subgroup analysis of chemotherapy regimens

Fourteen studies reported the chemotherapy regimens used in different cohorts, allowing patients to be grouped into the **R-CHOP**, **DA-EPOCH**, and **M/VCOP-B** groups. Heterogeneity among the groups was generally low (R-CHOP group: I² = 23.2%, *P* = 0.216; R-DA-EPOCH group: I² = 0%, *P* < 0.001; M/VCOP-B group: I² = 0%, *P* = 0.4), and thus a fixed-effects Mantel-Haenszel model was applied for analysis. The results of the subgroup analysis are presented in the forest plot (Fig. 4C). The **R-CHOP group** had a RR of 1.09 (95% confidence interval [CI]: 1.04–1.15), indicating a significant positive impact on PFS. The RRs for the **R-DA-EPOCH** and **M/VCOP-B** groups intersected the Line of no effect, suggesting no significant difference in PFS for these groups. This was further corroborated by subsequent Z-tests, with P-values greater than 0.05 for both groups (R-DA-EPOCH group: *P* = 0.079; M/VCOP-B group: *P* = 0.196).

### Publication bias

**Fig. 5:**
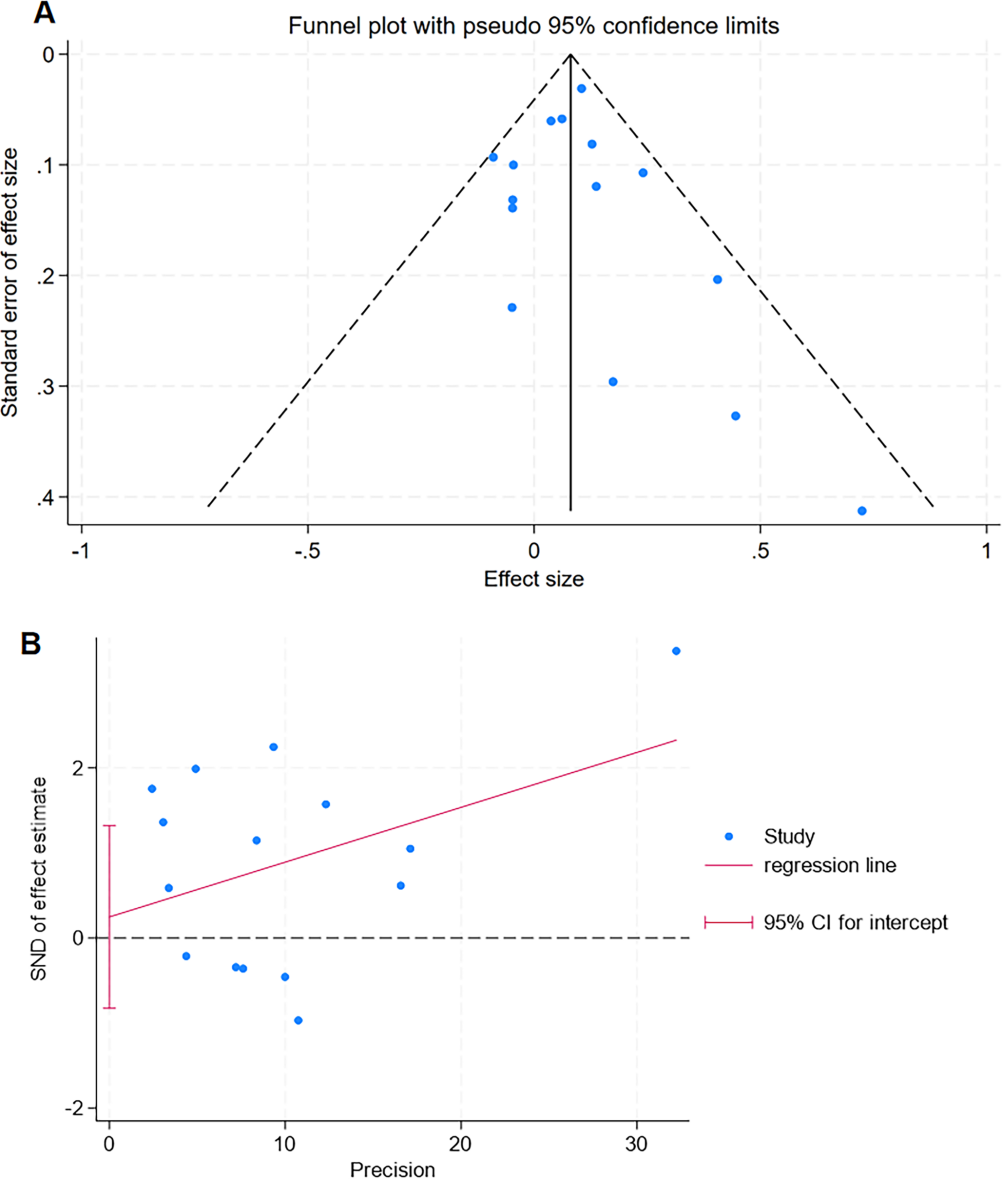
Publication bias of the 15 included studies. **A** Funnel plot constructed using the Harbord method. **B** Funnel plot constructed using the Egger test method

Publication bias was assessed using the Harbord method (Fig. 5A) and funnel plots constructed in combination with Egger’s test (Fig. 5B). Significant publication bias would be indicated if the P-values of both methods were less than 0.05.

Analysis of the 15 included studies revealed no evidence of publication bias (Egger’s test: t = 0.5, *P* = 0.627 > 0.05). Consequently, there was no need to apply the trim-and-fill method to evaluate the robustness of the pooled results.

### Sensitivity analysis

**Fig. 6:**
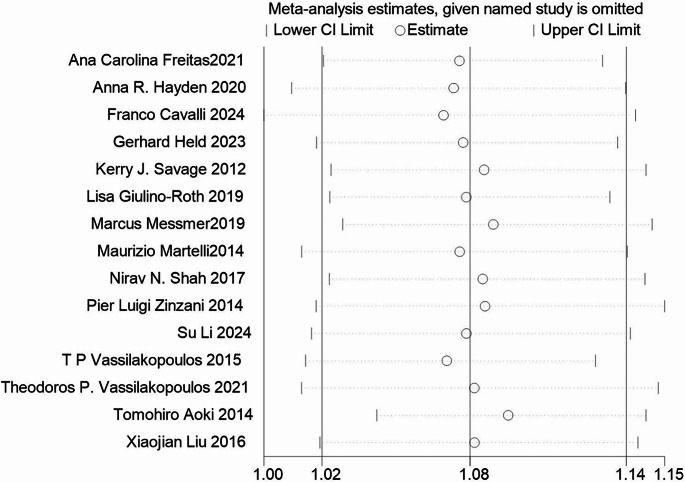
The graph of sensitivity analysis (the vertical line on the left indicated the total lower CI, the vertical line in the middle indicated the total pooled effect size, and the vertical line on the right indicated the total higher CI. The circle indicated the pooled effect size after deleting the study

A sensitivity analysis was conducted for all included studies, with the results presented in Fig. 6. The analysis revealed no significant heterogeneity among the 15 studies.

When each study was excluded sequentially, the remaining studies consistently produced RR values within the 95% confidence interval, indicating the robustness of the results. Figure 6 The graph of sensitivity analysis (the vertical line on the left indicated the total lower CI, the vertical line in the middle indicated the total pooled effect size, and the vertical line on the right indicated the total higher CI. The circle indicated the pooled effect size after deleting the study.

## Discussion

Patients with PMBCL who achieve CR after first-line immunochemotherapy generally have favorable outcomes without the need for additional radiotherapy. However, it remains unclear whether patients with non-CR following first-line therapy, when treated with consolidative radiotherapy, can achieve survival outcomes comparable to those of CR patients managed with observation alone. To address this question, we conducted a meta-analysis comparing survival outcomes between patients with non-CR receiving consolidative radiotherapy and those with CR managed with observation. Our results showed that radiotherapy significantly improved PFS in patients who did not achieve CR, although it did not lead to a statistically significant improvement in OS. Further subgroup analysis indicated that the PFS benefit of radiotherapy was more pronounced in patients treated with the R-CHOP regimen than in those receiving DA-EPOCH. These findings suggest that consolidative radiotherapy is beneficial and necessary for PMBCL patients with non-CR after initial treatment.

In contrast to the findings of the IELSG37 trial, which reported no added benefit of radiotherapy in patients achieving CR, our study suggests that radiotherapy may confer a PFS advantage in the non-CR setting. This discrepancy may be attributed to differences in study populations. While the IELSG37 trial evaluated patients in CR randomized to radiotherapy versus observation, our analysis focused on patients with residual disease who subsequently received radiotherapy. The differential therapeutic effects observed between CR and non-CR populations underscore the context-specific role of radiotherapy. Furthermore, due to incomplete demographic data, direct comparison of patient characteristics between our cohort and IELSG37 was not feasible. Another contributing factor to the observed divergence may Lie in the variation of chemotherapy regimens across studies. In our analysis, 62% of patients received the R-CHOP regimen (863/1392), compared to only 44% in the IELSG37 trial (161/364). Prior studies have suggested that more intensive regimens, such as DA-EPOCH, may reduce the need for consolidative radiotherapy [14, 36]. Our subgroup analysis supports this hypothesis, showing a significant PFS benefit of radiotherapy in R-CHOP–treated patients but not in those receiving DA-EPOCH or M/VCOP-B. However, the relatively small sample sizes in the latter groups may introduce bias and limit statistical power. To date, no randomized controlled trials have directly compared R-CHOP plus consolidative radiotherapy to DA-EPOCH, highlighting a key area for future investigation.

In this meta-analysis, no statistically significant difference in OS was observed between the radiotherapy and observation cohorts, which is consistent with the results of the pooled K-M curves. This finding may be partially explained by moderate heterogeneity across studies (I² = 60.3%, *P* = 0.014), potentially affecting the estimation of the treatment effect of radiotherapy. In addition, the overall favorable prognosis of PMBCL, reflected by 5-year survival rates exceeding 93% in both groups, may have attenuated the observable OS difference, despite the improved PFS.

Beyond efficacy, toxicity profiles and treatment feasibility are also critical considerations. DA-EPOCH has been associated with significantly higher rates of severe adverse events compared to R-CHOP, including Grade 4 neutropenia (90% vs. 56%), thrombocytopenia (35% vs. 6%), febrile neutropenia (37% vs. 19%), and neuropathy (18.6% vs. 3.3%) [37]. In addition, the combination stability of etoposide, vincristine, and doxorubicin in the DA-EPOCH regimen has been reported as suboptimal [38], increasing the complexity of administration. These Limitations have prompted exploration of alternative intensive regimens that balance efficacy and toxicity. For example, the R-COMP-DI regimen, which incorporates non-pegylated Liposomal doxorubicin, has demonstrated a CR rate of 93% without requiring hospitalization [39]. For patients ineligible for chemotherapy, emerging therapies such as Brentuximab and PD-1 inhibitors offer promising strategies to improve disease control and extend PFS.

This meta-analysis has several Limitations. First, as a meta-analysis rather than a prospective randomized controlled trial, it is subject to inherent methodological constraints. A majority of the studies did not report baseline data stratified by induction chemotherapy outcomes, preventing statistical analysis of demographic data across studies and Limiting the possibility of performing multivariate stratification. Secondly, of the 15 studies included, 12 were retrospective in design, and only three were prospective RCTs. The limited number of RCTs may undermine the robustness of the findings. Moreover, heterogeneity in treatment response assessment criteria across studies may introduce additional variability. Specifically, one study evaluated treatment response using CT, another employed the IHP criteria, while the remaining studies utilized the Deauville criteria. Such methodological inconsistency in response evaluation could affect the comparability of outcomes and the statistical robustness of the pooled analysis. Additionally, while the pooled Kaplan-Meier curves indicated a higher PFS rate in the radiotherapy group compared to the observation group, the log-rank test did not reveal a statistically significant difference between the two groups (*P* = 0.78, >0.05). This lack of significance may be attributable to the generally favorable prognosis of PMBCL, with a modest effect size between the radiotherapy and observation groups, insufficient to achieve statistical significance. Furthermore, because the median follow-up duration across studies was less than five years, the incidence of radiotherapy-induced secondary malignancies could not be adequately assessed or quantified. Lastly, this analysis relies on published aggregate data, and the absence of individual patient-level data, coupled with publication bias favoring positive results, may lead to an overestimation of the clinical benefit of consolidation radiotherapy in PMBCL.

In conclusion, our findings support the use of consolidative radiotherapy in PMBCL patients who do not achieve CR following first-line immunochemotherapy. Future large-scale, multicenter studies and randomized controlled trials are warranted to validate these results and further delineate the role of consolidative radiotherapy in this setting, thereby informing optimal treatment strategies.

## Data Availability

This analysis is a meta-analysis which overviewed and extracted data from previouspublished papers. All these papers can be found online.
